# Editorial: Metabolism of Fruit Volatile Organic Compounds

**DOI:** 10.3389/fpls.2022.873515

**Published:** 2022-06-02

**Authors:** Jinhe Bai, María J. Jordán, Jian Li

**Affiliations:** ^1^Horticultural Research Laboratory, USDA-ARS, Fort Pierce, FL, United States; ^2^Research Group on Rainfed Agriculture for Rural Development, Department of Rural Development, Oenology and Sustainable Agriculture, Murcia Institute of Agri-Food Research and Development (IMIDA), Murcia, Spain; ^3^Engineering and Technology Research Center, Beijing Technology and Business University, Beijing, China

**Keywords:** genotype, biotic, abiotic, development, ripening, plant hormone, flavor, aroma

Volatile organic compounds (VOCs) are essential for fruit flavor, and along with sugars and acids, play a key role in the perception and acceptability of fruits by consumers. Over 1,700 VOCs have been identified from 90 different plant families (Knudsen et al., 2006). Attending to the biosynthesis pathways, these components can be divided into several classes: terpenoids, phenylpropanoids/benzenoids, fatty acid derivatives, and amino acid derivatives (Figure 1). Volatile blends in plants are complex and differ among species and even cultivars (Barbey et al.; Colonges et al.; Deng et al.; Fan et al.; Zidi et al.). These volatile profiles can be affected by biotic and abiotic stresses (Sun et al.), plant hormone/regulator applications (Han et al.; Tobaruela et al.), and fruit mature/ripening stages (Gong et al.; Xin et al.). Some volatile compounds are not only important to aroma quality but also enhance or suppress sweetness perception in fresh fruit (Baldwin et al., 1998). Fan et al. identified 20 volatiles that enhance sweetness independently of sugars, such as benzaldehyde, (*E*)-2-pentenal, non-anal, mesifurane, ethyl butanoate, and ethyl hexanoate.

**Figure 1:**
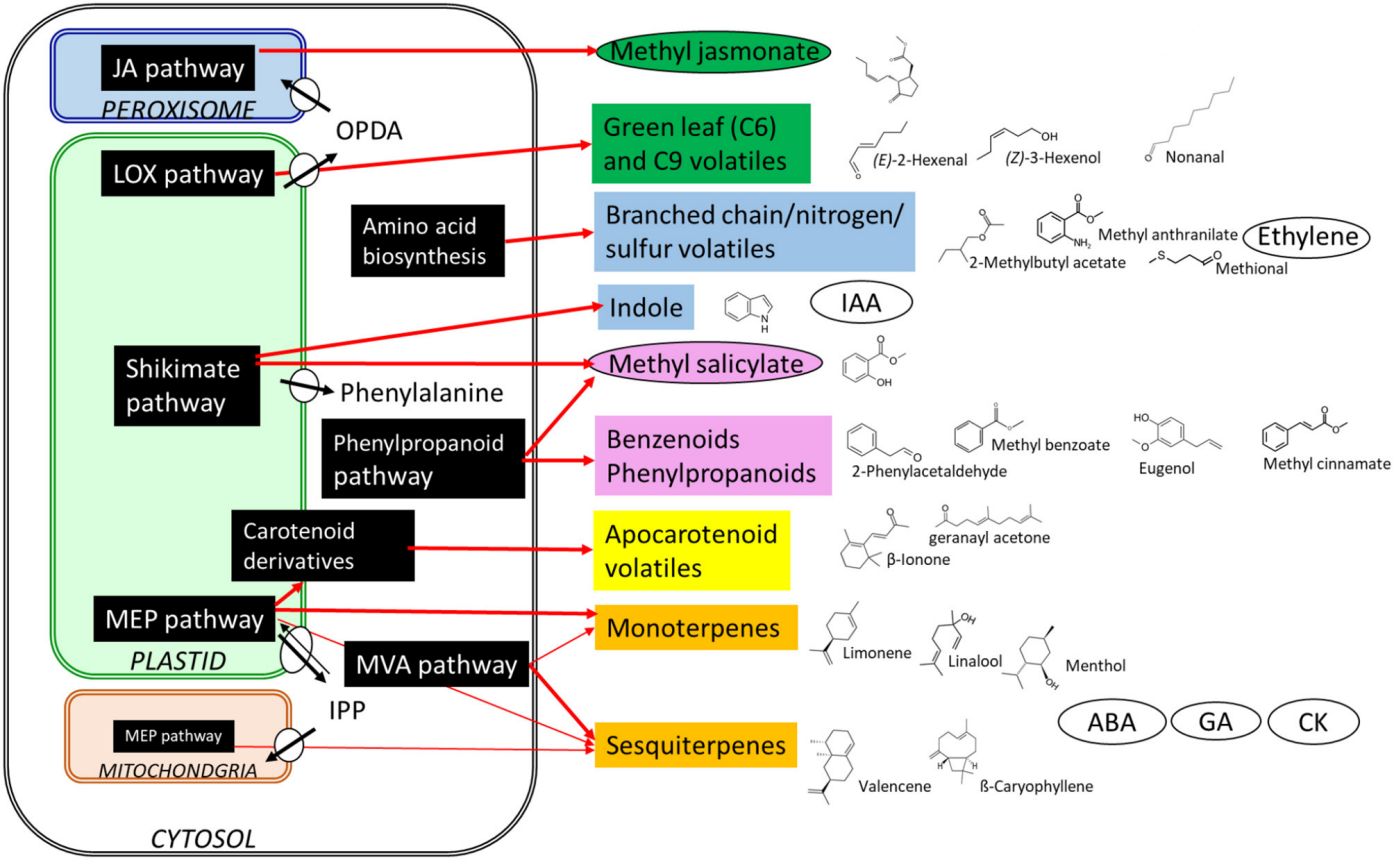
Biosynthesis of different volatile organic compounds and hormones in plants. Shawn are structures and the biosynthetic origin. MVA, mevalonic acid; MEP, methylerythritol phosphate; JA, jasmonic acid; LOX, lipoxygenase; OPDA, cis-(+)-12-oxo-phytodienoic acid; IPP, isopentenyl diphosphate; IAA, indole-3-acetic acid; ABA, abscisic acid; GA, gibberellic acid; CK, cytokinin. Plant hormones are in the oval frames.

In the scope of applications of molecular markers in VOC research, two studies go in-depth to identify putative candidate genes involved in major volatile biosynthesis pathways using a genome-wide association study (GWAS). Barbey et al. integrated GWAS, transcriptomic, and metabolomic analyses to identify QTL and candidate genes associated with multiple aroma compounds in strawberry breeding populations. Novel fruit volatile QTLs were discovered for methyl anthranilate, methyl 2-hexenoate, methyl 2-methylbutyrate, mesifurane, and a shared QTL on Chr 3 was found for nine monoterpene and sesquiterpene compounds. These QTLs present new opportunities in breeding for improved flavor in commercial strawberries. Colonges et al. studied the genetic and biochemical determinism of the floral aroma of the modern *Nacional cocoa* variety from Ecuador. GWAS was conducted on a population of 152 genotypes of cocoa trees using simple sequence repeats (SSR), single-nucleotide polymorphisms (SNP) markers, biochemical compounds (in roasted and unroasted beans), and sensory evaluations. The results showed that the monoterpene biosynthesis pathway and the L-phenylalanine degradation pathway are mainly related to the floral note.

A VOC profile is the key quality attribute in fruit, and is frequently compared between species, cultivars, and hybrids. Deng et al. identified 44 VOCs from 5 muscadine grape cultivars. The significant VOC differences among cultivars, particularly the bronze color and the purple-black color, are due to the quantitative differences of 29 volatiles. The following VOCs were characterized for each cultivar: geraniol and cinnamyl alcohol for Alachua, ethyl (*E*)-2-butenoate and propyl acetate for Noble, green leaf volatiles for Carlos, Fry, and Granny Val. Zidi et al. identified 29 VOCs from 3 fig cultivars. Two out of the three had 20 VOCs, but only 14 compounds in the third cultivar. Aldehydes comprised the most abundant VOCs identified, while alcohols, ketones, and terpenes comprised the minor compounds found in all cultivars. Specific VOCs in each cultivar were identified and those can be used as fingerprints to distinguish cultivars. Yang et al. identified 10 trans-isopentenyl diphosphate synthase (TIDS) genes, which are involved in terpene diversity and the accumulation of terpenoids, in the genome of the *Cinnamomum camphora* borneol chemotype that was unevenly distributed on chromosomes. Synteny analysis revealed that the TIDS gene family in this species likely expanded through segmental duplication events.

Changes in VOCs during fruit development and ripening are another relevant issue for the integration of transcriptomic and metabolomic levels. Gong et al. generated the metabolomic and transcriptomic datasets during watermelon fruit growth and development with a total of 517 metabolites. Major VOCs, such as (*E,Z*)-2,6-nonadienal, (*E*)-2-nonenal, and (*Z*)-3-nonen-1-ol, were accumulated during fruit development with sugar increase and acid decline. They found that five alcohol dehydrogenase (ADH) genes were differentially expressed which were closely related to C9 alcohol/aldehyde metabolism. The integration of important genes in the glycolytic and LOX pathways provides molecular insights into flavor formation. Xin et al. identified 181 VOCs from mongo fruits throughout the development and ripening stages. These components, especially ethanol and (*E,Z*)-2,6-nonadienal, were the key aroma active compounds, and raised during ripening. RNA sequencing revealed 53,384 transcripts in mango, and catalytic activity, transferase activity, adenosine diphosphate binding, transcription factor activity, and oxidoreductase activity were differentially expressed. The content of α-pinene and the expression of genes and enzyme activities in terpenoid metabolic pathways gradually increased after harvest and generally plateaued when reaching the ripe stage. The integrative analyses also revealed potential molecular insights into fruit ripening and aroma biosynthesis. Zidi et al. analyzed the changes of VOC profiles in three fig cultivars. Different aroma descriptors were identified during the postharvest fruit development stages; fruity and green aromas were dominant in all cultivars, while a fatty aroma barely occurred. They demonstrated that certain VOCs differentiate the ripening stages in figs.

Fruit ripening is associated with hormone interaction (McAtee et al., 2013), and a complicated change of VOC profile occurs during ripening. However, Tobaruela et al. provided direct evidence that ethylene and auxin affect VOC profiles independent of ripening. Two tomato cultivars, Micro-Tom and Sweet Grape, had different VOC profiles when reaching the ripening stage. However, both were similarly affected by exogenous ethylene and auxin treatments, with contrary responses to the hormones. Both hormones affect the aroma metabolism overlap, and the hormonal regulation is not cultivar-dependent. The hormones played essential roles in VOC metabolism, especially regarding carotenoid and fatty acid-derived compounds. Han et al. sprayed abscisic acid (ABA) at the late green stage of blueberries resulting in a substantial increase in anthocyanins. The direct reason for the result is due to the high expression of *CHS, CHI, DFR*, and *LDOX/ANS* during ripening. Among the transcription factors whose expression changed, *VcMYBA* had the greatest correlation with the changes of anthocyanin and corresponding synthetic structural genes.

Sun et al. demonstrated how biotic and abiotic factors impact VOC profiles in orange peel oil. Orange trees infected or not infected with huanglongbing disease were sprayed with fungicides/chemical agents. The infected fruit (HLB+) peel had lower concentrations of typical peel oil components, including valencene, octanal, and decanal, and was abundant in oxidative/dehydrogenated terpenes, such as carvone and limonene oxide. One of the enhanced foliar spray programs altered the VOC profile of HLB+ samples, resulting in peel oil similar to that of HLB- samples.

In summary, the work reported here gathered new information on the metabolism of VOCs at the genetic, transcriptional, hormonal, and metabolic levels and their impact on fruit flavor quality. We hope that this Research Topic will encourage scientists to make further efforts to reveal the mechanism of VOC changes and enhance fruit flavor quality.

## Author Contributions

All authors listed have made a substantial, direct, and intellectual contribution to the work and approved it for publication.

## Funding

This work was partially supported by the U.S. National Institute of Food and Agriculture (NIFA; Grant Number 2018-70016-27453).

## Author Disclaimer

The mention of trade names or commercial products in this publication is solely for the purpose of providing specific information and does not imply recommendation or endorsement by the U.S. Department of Agriculture. USDA is an equal opportunity employer.

## Conflict of Interest

The authors declare that the research was conducted in the absence of any commercial or financial relationships that could be construed as a potential conflict of interest.

## Publisher's Note

All claims expressed in this article are solely those of the authors and do not necessarily represent those of their affiliated organizations, or those of the publisher, the editors and the reviewers. Any product that may be evaluated in this article, or claim that may be made by its manufacturer, is not guaranteed or endorsed by the publisher.
